# Discovery and Targeting of the Signaling Controls of *PNPLA3* to Effectively Reduce Transcription, Expression, and Function in Pre-Clinical NAFLD/NASH Settings

**DOI:** 10.3390/cells9102247

**Published:** 2020-10-07

**Authors:** Brian E. Schwartz, Vaishnavi Rajagopal, Cynthia Smith, Evan Cohick, Gavin Whissell, Mario Gamboa, Rutuja Pai, Alla Sigova, Iris Grossman, David Bumcrot, Kavitha Sasidharan, Stefano Romeo, Alfica Sehgal, Piero Pingitore

**Affiliations:** 1 CAMP4 Therapeutics, Cambridge, MA 02139, USA; vaishnavi.rajagopal@gmail.com (V.R.); smith@camp4tx.com (C.S.); cohick@camp4tx.com (E.C.); whissell@camp4tx.com (G.W.); gamboa@camp4tx.com (M.G.); pai@camp4tx.com (R.P.); sigova@camp4tx.com (A.S.); iris@grossmail.com (I.G.); bumcrot@camp4tx.com (D.B.); sehgal@camp4tx.com (A.S.); 2 Department of Molecular and Clinical Medicine, University of Gothenburg, SE-413 45 Gothenburg, Sweden; Kavitha.Sasidharan@wlab.gu.se (K.S.); stefano.romeo@wlab.gu.se (S.R.)

**Keywords:** NASH, NAFLD, PNPLA3, momelotinib, CYT-387, ACVR1, stellate, JAK, BMP signaling, fibrosis, rs2294918

## Abstract

Non-alcoholic fatty liver disease (NAFLD) and non-alcoholic steatohepatitis (NASH) are emerging worldwide epidemics, projected to become the leading cause of liver transplants. The strongest genetic risk factor for NAFLD/NASH susceptibility and progression is a single-nucleotide polymorphism (SNP) in the patatin-like phospholipase domain-containing 3 gene (*PNPLA3*), rs738409, encoding the missense mutation I148M. This aminoacidic substitution interferes with the normal remodeling of lipid droplets in hepatocytes. It is also thought to play a key role in promoting liver fibrosis by inhibiting the release of retinol from hepatic stellate cells. Reducing *PNPLA3* levels in individuals homozygous for 148M may be an effective treatment for the entire spectrum of NAFLD, based on gene dosage analysis in the human population, as well as the protective effect of another naturally occurring SNP (rs2294918) in *PNPLA3* which, when co-inherited, reduces *PNPLA3* mRNA levels to 50% and counteracts disease risk. By screening a clinical compound library targeting specific signaling pathways active in primary human hepatocytes, we identified momelotinib, a drug evaluated in clinical trials to treat myelofibrosis, as a potent down-regulator of *PNPLA3* expression, across all genotypes. We found that momelotinib treatment yielded >80% reduction in *PNPLA3* mRNA in human primary hepatocytes and stellate cells, as well as in vivo via acute and chronic treatment of WT mice. Using a human multilineage 3D spheroid model of NASH homozygous for the PNPLA3 mutant protein, we additionally show that it decreases *PNPLA3* mRNA as well as intracellular lipid content. Furthermore, we show that the effects on *PNPLA3* coincide with changes in chromatin accessibility within regulatory regions of the *PNPLA3* locus, consistent with inhibition occurring at the level of transcription. In addition to its primary reported targets, the JAK kinases, momelotinib inhibits several non-JAK kinases, including ACVR1. Using a combination of targeted siRNA knockdowns and signaling pathway perturbations, we show that momelotinib reduces the expression of the *PNPLA3* gene largely through the inhibition of BMP signaling rather than the JAK/STAT pathway. Overall, our work identified momelotinib as a potential NASH therapeutic and uncovered previously unrecognized connections between signaling pathways and *PNPLA3.* These pathways may be exploited by drug modalities to “tune down” the level of gene expression, and therefore offer a potential therapeutic benefit to a high at-risk subset of NAFLD/NASH patients.

## 1. Introduction

The recognition of non-alcoholic steatohepatitis (NASH) as an emerging worldwide epidemic has added a new sense of urgency for the identification of novel targets and therapeutic modalities. This call for action, however, has been tempered by the growing realization that non-alcoholic fatty liver disease (NAFLD)/NASH represents a complex disease spectrum involving the interaction of several cell types, signaling networks, and genetic variants. In general, individual single nucleotide polymorphisms (SNPs) have only a weak influence on phenotype, and are largely nonpredictive for disease outcomes unless aggregated [1,2]. A notable outlier is the prevalent rs738409 SNP (minor allele frequency of 0.17–0.49, depending on ethnic origin) in the coding region of the patatin-like phospholipase domain-containing 3 (*PNPLA3*) gene that encodes the missense variant I148M [3]. Though *PNPLA3* had been studied since 2001, a landmark genome-wide association study in 2008 revealed *PNPLA3* rs738409 as the strongest genetic risk factor for hepatic fat and inflammation across ethnicities, and refocused attention on this gene for its suspected role in NASH [3,4]. The subsequent decade saw numerous independent reports confirming the initial finding and expanding on the remarkable odds ratios for disease susceptibility (OR = 5.05 in homozygous individuals), rendering it a particularly attractive novel target for therapeutic intervention [5,6]. In addition, dozens of studies have been conducted to elucidate the biology of this protein and how a single point mutation can be associated with such a robust effect on the progression of NAFLD/NASH.

The totality of evidence indicates that the wild-type PNPLA3 protein resides in the membrane of lipid droplets, where it is responsible for the postprandial remodeling of lipid droplets via its triglyceride hydrolase activity. PNPLA3 I148M has 80% less catalytic activity, but the more critical consequence of this mutation (and another catalytically impaired version, S47A) is its resistance to ubiquitylation-based degradation [7,8]. The result is an accumulation of PNPLA3 148M protein on the surface of lipid droplets, which then sequesters CGI-58, a cofactor required for the activity of adipose triglyceride lipase (ATGL) [9]. The net effect is an impairment of lipase activity and increased droplet size, which manifests as hepatic steatosis.

*PNPLA3* is also abundantly expressed in hepatic stellate cells (HSCs) in both humans and mice, where its retinyl hydrolase activity facilitates the release of retinol from lipid droplets [10]. The variant PNPLA3 148M has reduced hydrolase activity, and has been shown to promote the production of pro-fibrogenic cytokines, including CCL2 and CCL5, which stimulate HSC activation and therefore additionally promote inflammation and fibrosis in NAFLD/NASH [10,11,12]. HSCs are responsible for the secretion of collagen 1, a major collagen protein that forms the basis of fibrosis during liver injury, as occurs in late stages of NASH. The silencing of *PNPLA3* in 148M knock-in mice with antisense oligos (ASOs) reduced liver collagen [13]. Therefore, reducing the expression of *PNPLA3* 148M in HSCs in addition to hepatocytes is expected to provide a multi-faceted therapeutic benefit for NASH patients.

Here, we identify a novel mechanism for the control of *PNPLA3* transcription, expression, and subsequent function. We further characterize a novel mode of action for momelotinib that is responsible for its potent downregulation of *PNPLA3* expression in both primary human hepatocytes and primary HSCs. We initially uncovered it in a focused screen of clinical-stage small molecules targeting specific cell signaling pathways known to be active in primary human hepatocytes and consistently modulate *PNPLA3*. Though at high doses momelotinib was designed to treat myeloproliferative neoplasms harboring JAK2 mutations, it has activity against other kinases, including the receptor serine kinase, ACVR1 [14,15,16]. Using a series of chromatin-based assays and combinatorial siRNA knockdown experiments, we show that it is primarily through the inhibition of the ACVR1 pathway that momelotinib decreases *PNPLA3* at the level of transcription. Furthermore, the treatment of primary hepatocytes bearing *PNPLA3* rs738409 (148M variant) results in lipid reduction, consistent with the alleviation of PNPLA3 148M-induced steatosis. We also show that momelotinib suppresses the TGFβ-induced transcription of several profibrotic and proinflammatory genes in primary HSCs, representing yet another potential route to combat NAFLD/NASH progression.

A 3D multilineage in vitro system that has previously been used to model NASH was then employed and functionally confirmed our results. This model, developed by Pingitore et al., comprises *PNPLA3* 148 M/M immortalized hepatocyte and stellate cells, allowing it to replicate simple steatosis as well as progression to fibrosis in response to fatty acid loading. Furthermore, it accurately recapitulates the anti-steatotic and anti-fibrotic properties of elafibranor, a PPARα/δ inhibitor currently in phase 3 clinical trials for NASH, therefore demonstrating its predictive value [17]. Lastly, we show that momelotinib achieves the target effect size for expected therapeutic benefit, reducing hepatic *PNPLA3* mRNA in vivo by over 50% when administered to wild-type mice fed a high-sucrose diet.

Overall, our work reveals new signaling pathways that control *PNPLA3* transcription, and highlights momelotinib as a drug candidate for treating a substantial genetically defined subpopulation of NAFLD/NASH patients.

## 2. Materials and Methods

### 2.1. Animals

C57BL/6 males from Taconic Biosciences at 7–9 weeks of age were housed three animals per cage and kept on a 12 h light/dark cycle with access to food only during the dark. On Day 1 of the study, they were switched to a high-sucrose diet (no. 901,683 from MP Biomedicals, Santa Ana, CA, USA) and assigned into groups of *n* = 6. Mice were allowed access to a high-sucrose diet only during the 12 h dark cycle for the full duration of the 10-day experiment to metabolically synchronize their PNPLA3 levels. Momelotinib was administered QD in the evening via oral gavage at a volume of 5 mL/kg starting on Day 7 and continuing until Day 11. Mice were sacrificed on the morning of Day 11 within 1 h of food removal. All vivarium experiments were performed at Hooke Laboratories (Lawrence, MA, USA). At the end of the study, the mice were sacrificed within 1h of the last dose. Liver tissue was harvested and total RNA was extracted for qPCR analysis. This study (approval code PR20180312-3) was performed under protocol 1701PK-Tolerance (v1), approved 9 January 2017 (currently 1701PK-Tolerance (v2), approved 12 September 2019). All procedures were performed within the guidelines of the Institutional Animal Care and Use Committee (IUCUC).

### 2.2. RNA Expression and Real-Time PCR

Total RNA from primary human hepatocytes and stellate cells was isolated using the RNeasy Kit from Qiagen (cat#74181) and converted to cDNA with the High-Capacity cDNA Reverse Transcription Kit from ThermoFisher Scientific (cat#4368813). All experiments were performed in triplicate using a housekeeper control. Quantitative real-time PCR was run on a One Step Plus thermocycler from Applied Biosystems, and relative gene expression was calculated from the threshold cycle (Ct) numbers using the ΔΔCt method. For mouse liver samples, the tissue was flash frozen after harvest, and pulverized to a powder. Approximately 2–5 mg were dissolved in Trizol reagent (Sigma), and RNA was purified using RNeasy 96-well blocks. The on-column DNAse step was included. All samples were normalized to 50 ng/μL based on 260/280 readings, and 500 ng total RNA were used for cDNA synthesis, as above.

### 2.3. Genotyping Assays for Primary Cells

The presence of *PNPLA3* rs738409 was detected using the Taqman Predesigned SNP Genotyping Assay from Applied Biosystems (cat#4351379) according to the manufacturer’s instructions.

### 2.4. Lipid Droplet Staining of Primary Hepatocytes

The lipid droplets were stained using oil red O (ORO) and the nucleus with methyl green as per the manufacturer’s protocol (BioVision, Cat #K584). Briefly, the treated cells were washed gently with PBS and then fixed with 10% formalin. The fixed cells were then washed twice with water and permeabilized using 60% isopropanol. The cells were then incubated with ORO solution for 20 min with gentle shaking on an orbital shaker. Excess stain was removed by washing 3–5 times with water. The nuclei were stained with a brief incubation with methyl green. Excess stain was removed by washing 3–5 times with water. Cells from triplicate wells were imaged using light microscopy. For quantitation of ORO staining, Image J software was used as per the user manual (user guide IJ1.46r). Briefly, the RGB TIFF images were converted to 8-bit images and then set to binary image to highlight the stained particles and to enable quantitation of staining intensity. The analyze menu was then used to measure average integrated density of all the highlighted particles in each image.

### 2.5. Human Primary Hepatocyte Cell Culture

Primary human hepatocytes obtained from Massachusetts General Hospital (Boston, MA, USA), In Vitro ADMET Laboratories (Columbia, MD, USA), or Lonza (Switzerland) were plated in collagen-coated wells as per the manufacturers’ instructions and incubated at 37 °C and 5% CO_2_ for 4 h to allow attachment. For drug treatments, momelotinib (SelleckChem, cat# S2219) was diluted in DMSO and added to hepatocytes in complete medium for 18 h at 37 °C and 5% CO_2_ prior to takedown. The same volume of DMSO was used in control wells. In experiments where BMP2 was used, BMP2 (R&D Systems, Inc., Minneapolis, MN, USA, cat# 355-BM-100) was reconstituted in 0.1% BSA/4 mM HCl to 100 μg/mL. It was used at a final concentration of 6.2 μg/mL for 18 h. For siRNA knockdown, primary hepatocytes were reverse transfected using RNAiMax (Invitrogen, cat# 13778-075) with 10 nM siRNA from Dharmacon (siGENOME). Following an overnight transfection in hepatocyte plating medium (Lonza, cat# MP100), the medium was replaced with complete medium and cultured for an additional 48 h, for a total of 72 h of knockdown.

### 2.6. Primary Human Hepatic Stellate Cell Culture

All primary HSCs (pHSCs) were obtained from ScienCell (Carlsbad, CA, USA). *PNPLA3* 148 I/I (cat#5300, lot 16604) cells were derived from a 15 yo female. *PNPLA3* 148 M/M cells (cat#5300, lot 20091) were derived from a female at 23 weeks’ gestation. The pHSCs were cultured in ScienCell stellate cell medium (cat#5301) on poly-d-lysine-coated plates as per SciencCell protocol. Cells at passage number 3 or 4 were used for experiments. For analysis of profibrogenic and proinflammatory gene expression, cells were incubated with 100 ng/mL TGFβ for 18 h.

### 2.7. Spheroid Cell Lines

HepG2 cells were purchased from the ATCC (Menassas, VA, USA). Immortalized human hepatic stellate cells (LX-2) were purchased from Millipore (Burlington, MA, USA). HepG2 and LX-2 cells were grown and maintained as previously described [17]. HepG2 and LX-2 cells were genotyped for *PNPLA3* rs738409 and confirmed homozygosity for the 148M mutant protein allele variant.

### 2.8. 3D Spheroid Culture

For the generation of the cell spheroids, cells were seeded into 96-well round-bottom ultra-low attachment plates (Corning) at 2000 viable cells per well. HepG2/LX-2 24:1 spheroids were grown in minimum essential medium (MEM) supplemented as previously described [17]. The volume of spheroids was determined using the following formula: 4/3 π r^3^, where “r” is the mean of the long diameter and short diameter of the spheroid divided by two.

### 2.9. Induction of Steatosis

Palmitic acid, oleic acid, and bovine serum albumin (BSA) were purchased from Sigma-Aldrich (St. Louis, MO, USA). After spheroid aggregation 72 h after seeding, HepG2/LX-2 24:1 spheroids were exposed to a mixture of fatty acids palmitic acid and oleic acid 500 μM (1:2) conjugated to BSA for a further 48 h, after which spheroids were collected. For all the tested conditions, media were supplemented with 1% bovine serum albumin (BSA). For conjugation, fatty acids were dried under nitrogen flow and resuspended in medium (1/10 of the desired volume) containing 10% BSA and mixed overnight at 40 °C. The day after, medium was diluted 1:10 with fresh medium and used to treat the spheroids.

### 2.10. Drug Treatments for Spheroids

Pre-steatotic spheroids were subject to drug treatment. The preventive effect of steatosis was assessed with momelotinib (1, 5 and 10 μM). Momelotinib was purchased from Selleck Chemicals.

### 2.11. Lipid Assay in Spheroids

AdipoRed assay reagent (Lonza, Basel, Switzerland) was used to measure lipid accumulation according to the manufacturer’s instructions. Briefly, spheroids were collected and moved to a new 96-well clear-bottom plate with 200 μL of PBS in each well. Twenty microliters of trypsin were added and incubated at 37 °C for 20 min. Next, 7 μL of AdipoRed reagent were added in each well, mixed by pipetting, and incubated for 10 min at room temperature. The fluorescence was analyzed by a SpectraMax i3 (Molecular Devices, San Jose, CA, USA) counter.

### 2.12. Oil Red O Staining

The 3D spheroids were fixed with 10% (w/v) paraformaldehyde (PFA, Sigma-Aldrich) for 2 h, then incubated with 20% (w/v) sucrose in phosphate-buffered saline (PBS, Lonza) overnight, washed three times with PBS, embedded in OCT cryomount (Histolab, Västra Frölunda, Sweden), and stored at −80 °C. Spheroids were sectioned into 8 µm thick slices using a cryostat (Leica, Wetzlar, Germany). Sections were stored at −80 °C. The total area of ORO-stained lipid droplets was determined as previously described [18]. Briefly, lipid droplets were stained by oil red O staining solution (Sigma-Aldrich) by dissolving ORO in isopropanol (0.5%) and then mixing three parts of oil red O solution with two parts of distilled water. Nuclei were stained by hematoxylin or DAPI. Images with hematoxylin-stained nuclei were obtained using an Axio Imager M1 (Zeiss, Oberkochen, Germany) and AxioVision 4.8 software (Zeiss, Oberkochen, Germany), while images with DAPI-stained nuclei were obtained by an Axioplan 2 (Zeiss) using AxioVision 4.8 software (Zeiss).

### 2.13. Chromatin Immunoprecipitation (ChIP) Assays

Hepatocytes in HCM medium (Lonza, Basel, Switzerland) were treated for 16 h with 10 μM momelotinib in the presence of BMP2 (6.2 μg/mL). ChIP assays were performed based on a protocol previously described [19]. The qPCR primers were designed according to the location of H3K27Ac and H3K4me1 peaks from ENCODE data. The primer sequences were as follows: Primer 1 F: TTCCAAGAGCCAGCAAACGGG, Primer 1 R: GCAGGGACGGATGGGAAGAAG; Primer 2 F: GCCATCTTGAGTGTGGCAGGG, Primer 2 R: GGCCCAACAACCTTGACTCCA; Primer 3 F: ATTCCTGCCAGGGGCAATGTC, Primer 3 R: ATGTCCCTAAGGGCAGCCCAA; Primer 4 F: AGGCCTGTTGACACCCTCAGA, Primer 4 R: GTGATCCCCTCCCCCACTCTT; Primer 5 F: ATTCAAGGCCATGTCCCCAGC, Primer 5 R: GGAGCAAACCCCAGCTGAACT. Negative Control Primer F: GCCGCGCTTCAACAACAACTT, Negative Control Primer R: TTAATTCGGGACGCCTGGGTG.

### 2.14. Assay of Transposase-Accessible Chromatin (ATAC) Assays

Hepatocytes in HCM medium (Lonza, Basel, Switzerland) were treated for 1 h or 16 h with 10 μM momelotinib in the presence of BMP2 (6.2 μg/mL). ATAC assays were performed based on a protocol previously described [20]. The same qPCR primers used for ChIP-qPCR were used for ATAC-qPCR.

### 2.15. Statistical Analysis

Data from in vitro experiments were analyzed using unpaired *t*-tests or by one-way ANOVA. *p*-values of <0.05 were considered significant and are indicated as * in figures, or if <0.01 as **. *p*-values of 0.001 are indicated as ***, and *p* < 0.001 are indicated as **** in figures. Bar graphs in figures show mean ± SD of at least three experiments unless specified otherwise.

## 3. Results

### 3.1. Pathway-Tailored Perturbations Identify Momelotinib as a Potential Regulator of PNPLA3 Expression

**Figure 1:**
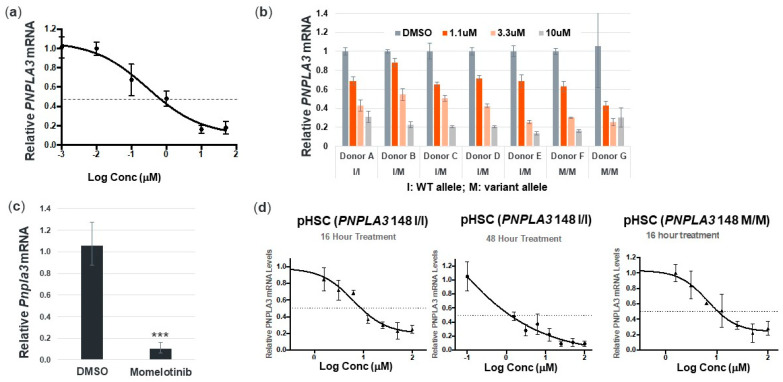
Momelotinib treatment reduces patatin-like phospholipase domain-containing 3 (*PNPLA3*) expression dose dependently in human primary hepatocytes and stellate cells. (**a**) Dose–response curve for *PNPLA3* mRNA in primary human hepatocytes (pHHs) after 16 h of treatment with momelotinib; (**b**) dose-dependent reduction of *PNPLA3* mRNA in pHHs representing different PNPLA3 codon 148 genotypes. I: WT allele, M: variant allele; (**c**) *Pnpla3* mRNA in mouse primary hepatocytes treated with 10 μM momelotinib or DMSO control for 18 h; (**d**) dose response curve for *PNPLA3* mRNA in WT primary human stellate cells (pHSCs) after 16 h and 48 h of treatment with momelotinib. First two panels are WT pHSCs, and the third panel is *PNPLA3* 148M/M pHSCs. All cell culture experiments were performed in triplicate, and the bars represent the mean of triplicate experiments. Error bars represent the SD of the triplicates. An unpaired *t*-test was used for panel (**c**), where *p* < 0.001.

To identify compounds that can inhibit the expression of *PNPLA3* in human primary hepatocytes, we assembled a panel of 18 clinical-stage small molecule inhibitors targeting specific signaling pathways that are known to be active in these cells. Testing was enriched for clinically validated agents alongside class members with semi-overlapping selectivity. Momelotinib emerged as a strong inhibitor of *PNPLA3* expression and reduced *PNPLA3* mRNA in a dose-dependent manner in human primary hepatocytes derived from a healthy donor. Over the course of an 18 h treatment, *PNPLA3* mRNA was reduced up to 80% at 10 μM, without affecting cell viability (Figure 1a). Of note, 10 μM is roughly five-fold lower than the peak plasma concentration achieved with a single 150 mg oral dose of momelotinib in a dose escalation study in metastatic cancer patients [21].

### 3.2. Momelotinib Reduces PNPLA3 in Human Hepatocytes and Stellate Cells from Multiple Donors, as well as Mouse Hepatocytes

To confirm the effect of momelotinib on *PNPLA3* more broadly, we tested primary hepatocytes from seven additional human donors, representing a range of *PNPLA3* SNP rs2294918 genotypes, across both genders. Regardless of gender or genotype, momelotinib effectively decreased *PNPLA3* mRNA in a concentration-dependent manner up to 85%, with an average EC50 of 1.8 μM (Figure 1b). We also confirmed that 10 μM momelotinib reduced *Pnpla3* levels by >80% in mouse hepatocytes (Figure 1c), demonstrating that this gene regulatory mechanism is conserved across species.

HSCs play a key role in the progression and pathogenesis of NAFLD/NASH. *PNPLA3* upregulation contributes to HSC activation, and in carriers of the 148M variant, there is an increased release of proinflammatory and profibrogenic cytokines [11,12]. It has also been further demonstrated that in HSCs, retinol release is dysregulated by PNPLA3 148M, which further enables excessive hepatic fibrosis in the context of NASH [10]. We therefore evaluated whether momelotinib could reduce *PNPLA3* expression in primary HSCs. We indeed observed a concentration-dependent reduction in mRNA that exceeded 50% within 16 h at 10 μM (Figure 1d, first panel). Further reductions of over 80% could be attained at the same concentration after a 48h treatment (Figure 1d, second panel). These results, obtained in WT cells, were replicated in primary HSCs from a 148 M/M donor (Figure 1d, third panel). These results suggest that the cell signaling mechanisms controlling *PNPLA3* gene expression are similarly affected by momelotinib in hepatocytes and stellate cells, and potentially represent an added therapeutic advantage to using momelotinib to treat NASH.

### 3.3. Momelotinib Reduces Lipid Droplet Content In Vitro

**Figure 2:**
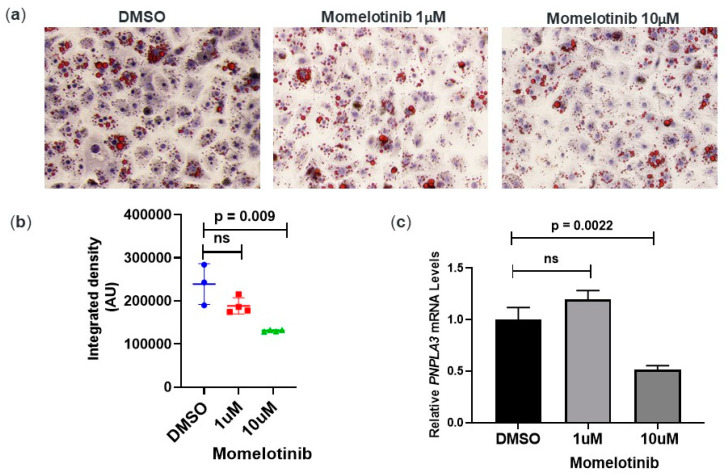
Momelotinib reduces neutral lipid staining in pHHs (148M/M). (**a**) Representative image of pHHs treated for 18 h with DMSO or momelotinib, 1 μM and 10 μM. Intracellular lipid droplets were visualized by oil red O (ORO) staining. Nuclei were counterstained in blue. (**b**) The average intensity of ORO staining was determined with Image J software using 3–4 images per treatment. An unpaired *t*-test was performed on resulting data. (**c**) Corresponding reduction in *PNPLA3* mRNA was determined by qPCR. Average values from triplicate wells are shown, along with SD. Only the 10 μM momelotinib treatment resulted in a significant reduction of *PNPLA3* mRNA, based on one-way ANOVA.

The presence of the *PNPLA3* 148M variant is associated with hepatic steatosis in GWAS population studies [3] and the lowering of *PNPLA3* expression with antisense oligonucleotides has been shown to reduce hepatic fat in rodents [13,22]. We therefore tested whether momelotinib treatment could reduce lipid droplet content in *PNPLA3* 148M/M primary human hepatocytes. For this, we treated monocultures of primary hepatocytes for 18 h with DMSO or momelotinib, followed by staining with oil red O (ORO) to visualize lipid content. At 10 μM momelotinib, there was a noticeable reduction in overall ORO staining, which we confirmed using imaging software to quantify several images for each treatment (Figure 2b). In agreement with this, we found a statistically significant 50% reduction in *PNPLA3* mRNA compared to DMSO (Figure 2c), potentially as a result of PNPLA3 reduction.

### 3.4. Momelotinib Reduces PNPLA3 mRNA In Vivo

**Figure 3:**
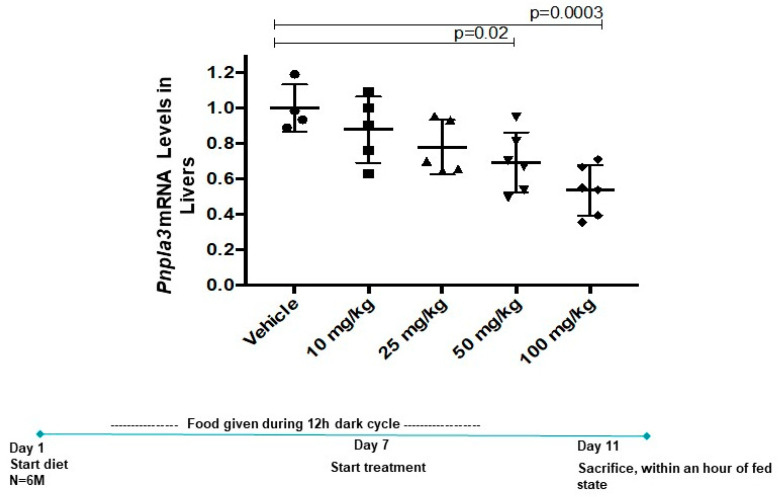
Momelotinib reduces *Pnpla3* mRNA dose dependently in vivo. Hepatic *Pnpla3* mRNA levels in WT mice administered five daily doses of momelotinib by oral gavage. *Pnpla3* levels were first metabolically synchronized by providing mice access to a high sucrose diet only during the dark cycle, depicted in the scheme below the graph. Six animals were used per treatment group; mean and SD are shown. Significance was determined using one-way ANOVA.

In a mouse model of myeloproliferative neoplasms (MPNs), momelotinib treatment resulted in the normalization of white blood cell counts, spleen size, and hematocrit by a multi-dose oral regimen of 50 mg/kg [15]. Because the MPN mice express an activating allele of JAK2, it is likely that momelotinib’s efficacy in this model is through JAK inhibition. To evaluate the effect of momelotinib treatment on *Pnpla3* expression in mouse livers, we gave mice restricted access (12 h per day only) to a high-sucrose diet for 6 days prior to the start of drug dosing. While this feeding regimen is not expected to produce a fatty liver phenotype within this timeframe (nor do these mice carry *Pnpla3* 148 M), restricted, high-sucrose feeding does serve to metabolically synchronize *Pnpla3* induction, allowing us to minimize variability within groups and make more precise measurements of the effect of the drug on gene transcription. Starting on Day 7, we administered five daily doses ranging from 10–100 mg/kg. A dose-responsive, 50% decrease in hepatic *Pnpla3* mRNA levels was achieved without signs of toxicity, a reduction that is predicted to have a measurable clinical benefit in NASH patients (Figure 3).

### 3.5. Momelotinib Reduces PNPLA3 mRNA and Triglycerides in a Novel In Vitro NASH Model

**Figure 4:**
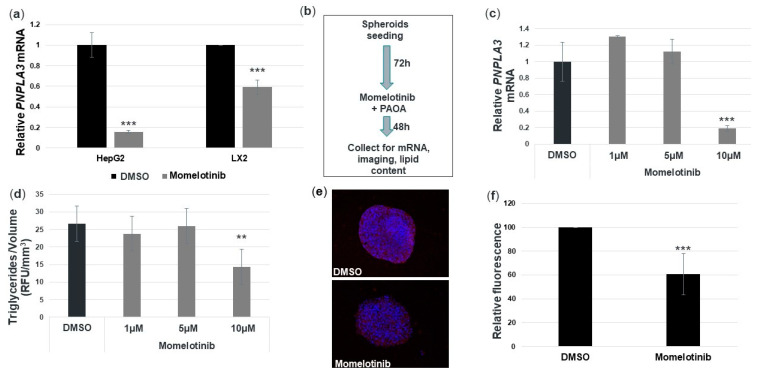
Momelotinib decreases *PNPLA3* mRNA in a 3D non-alcoholic steatohepatitis (NASH) model, composed of spheroid cultures of HepG2 and LX-2 cells. (**a**) *PNPLA3* mRNA is decreased in HepG2 and LX2 monolayer cultures after 18 h of treatment with 10 μM momelotinib. The average and SD of three independent HepG2 experiments and six independent LX2 experiments are shown. An unpaired *t*-test indicates statistical significance (*p* < 0.001). (**b**) Experimental overview of HepG2/LX2 spheroid formation and compound treatment. After allowing spheroids to form for 72 h, they were supplemented with a palmitic acid–oleic acid mixture (PAOA) along with momelotinib. After another 48 h, they were collected for imaging and RNA analysis. (**c**) *PNPLA3* mRNA levels after treatment of spheroids with momelotinib. The average of four independent experiments is shown with SD. An unpaired *t*-test shows a significant reduction with 10 μM momelotinib versus DMSO (*p* < 0.001). (**d**) Average triglyceride content after treatment with momelotinib, as assayed by relative fluorescence unit (RFU) and adjusted to the average spheroid volume (RFU/mm^3^). Shown is the average of five independent experiments along with SD. An unpaired *t*-test was used to calculate significance of 10 μM treatment relative to DMSO (*p* < 0.01). (**e**) Representative images of spheroids stained with DAPI (blue) and ORO (red) after treatment with momelotinib. (**f**) The relative fluorescence (arbitrary units) was analyzed for three independent spheroids calculated as ORO stain per number of nuclei. An unpaired *t*-test (**a**,**f**) or one-way ANOVA (**c**,**d**) indicate statistical significance.** indicates *p* ≤ 0.01; *** indicates *p* ≤ 0.001.

**Figure A1:**
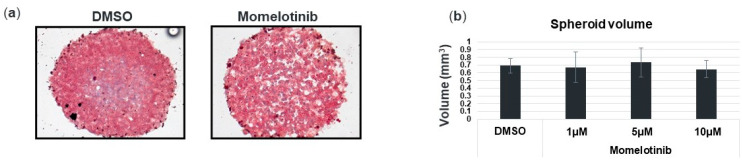
Spheroid volume was not affected by momelotinib treatment. (**a**) ORO stain of representative spheroid exhibits decreased lipid content after a 48hr momelotinib treatment compared to DMSO. Spheroids were primed with a palmitic–oleic acid mixture at the same time either momelotinib or DMSO was added. (**b**) Spheroid volume was calculated and averaged (see methods) to determine if viability or cell growth was affected by compound treatment.

NAFLD/NASH is widely acknowledged as a metabolic disease involving interactions among multiple hepatic cell types. Furthermore, our therapeutic approach to reducing PNPLA3 is unique in this class as it is the only therapeutic solution that targets both hepatocytes and HCSs simultaneously, holistically addressing the main culprits of disease pathoetiology. Therefore, we wanted to expand our studies beyond primary hepatocyte monocultures and test the efficacy of momelotinib in an in vitro disease model. For this, we used a mixed lineage 3D spheroid system that has been shown to reproduce aspects of steatosis and fibrosis. It consists of HepG2 cells combined with an immortalized stellate cell line, LX-2, both homozygous for *PNPLA3* 148M. It has been previously shown that this system can reproduce the fat reduction and anti-fibrotic effects of elafibranor, a phase 3 PPARα/δ inhibitor being developed for NASH [17]. We first tested momelotinib on monocultures of HepG2 and LX-2 cells and found that we could reduce *PNPLA3* mRNA by 80% and 40%, respectively, with a 10 μM concentration (Figure 4a). The experimental overview for the spheroid model experiments is shown in Figure 4b. We found that momelotinib induced an 80% reduction in *PNPLA3* mRNA at 10 μM (Figure 4c). Such a reduction is more similar to that observed in HepG2 monocultures than LX-2 monocultures, and may reflect the fact that the spheroids are composed of HepG2/LX-2 in a 24:1 ratio. A fluorometric assay was used to quantify the lipid content of the spheroids, and this value was normalized to the average spheroid volume for each treatment. Momelotinib reduced triglycerides by about 35% relative to the DMSO control (Figure 4d). A representative image of spheroids treated with DMSO or momelotinib exhibits a clear reduction in ORO staining consistent with the fluorometric assay (Figure 4e and Figure A1a in Appendix A). To confirm, we quantified several ORO-stained spheroid images from Figure 4e, and found that, compared to DMSO, there is indeed a 40% reduction in lipid staining. Spheroid volume was measured as a readout for cytotoxicity of the compound treatments (Figure A1b). We conclude that momelotinib is a potent reducer of fat content, as well as *PNPLA3* mRNA levels.

### 3.6. Momelotinib Reduces Expression of Profibrotic and Proinflammatory Genes in Primary Human Hepatic Stellate Cells

**Figure 5:**
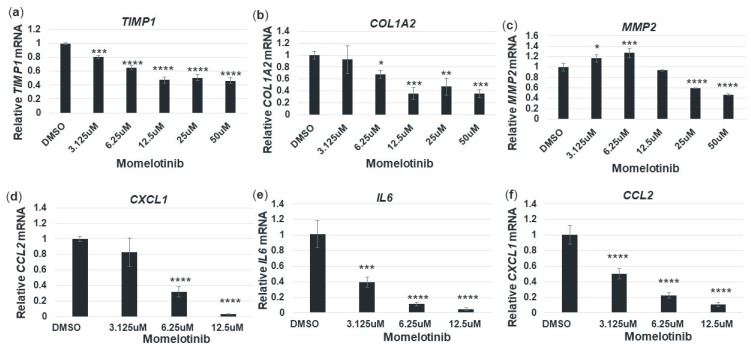
Momelotinib decreases the expression of profibrotic and proinflammatory genes in primary HSCs. Cells derived from a homozygous *PNPLA3* 148M donor were treated with either DMSO or momelotinib for 18 h. The cDNA was generated and used for qPCR to quantify gene expression of profibrotic genes *TIMP1*, *COL1A2*, and *MMP2* (**a**–**c**), as well as proinflammatory genes CXCL1, IL6, and CCL2 (**d**–**f**). Experiments were performed in triplicate and a one-way ANOVA was used to determine significance. Average and SD values are shown. *, **, ***, **** indicate *p* ≤ 0.05, *p* ≤ 0.01, *p* ≤ 0.001, and *p* ≤ 0.0001, respectively.

Independent lines of evidence indicate that reducing *PNPLA3* 148M can also reduce the expression of profibrotic genes and have a favorable impact on liver inflammation and fibrosis. For example, Linden et al. [13] have shown that ASO-mediated silencing of *Pnpla3* reduced liver inflammation scores and fibrosis stage in the *Pnpla3* 148M knock-in, but not WT mice. Similarly, Pingitore et al. [12] found that LX-2 cells stably overexpressing *PNPLA3* 148M (but not *PNPLA3* 148I) have increased expression of matrix metallopeptidase 2 (MMP2), and tissue inhibitor of metalloproteinase 1 and 2 (TIMP1 and TIMP2). To determine if momelotinib treatment could also reduce the expression of profibrotic genes in primary HSCs, we treated monocultures of *PNPLA3* 148M/M stellate cells with DMSO or momelotinib for 18 h in the presence of TGFβ. We found that for all genes tested, there was a clear reduction at the mRNA level, indicating a role for this drug in suppressing the TGFβ-induced expression of key profibrogenic markers (Figure 5a–c).

Hepatic stellate cells secrete proinflammatory cytokines in response to liver injury, which triggers the recruitment and infiltration of monocytes. In the spectrum of NAFLD/NASH, this is evident at the histological level, where it manifests as lobular inflammation, a component of the clinical NASH activity score (NAS). We therefore were interested in whether momelotinib could reduce the expression of some representative cytokines in TGFβ-treated pHSCs. Figure 5d–f show that all three genes tested were strongly repressed by momelotinib. These effects, as well as the effects on profibrotic gene expression, may be indirectly related to the reduction of *PNPLA3* 148M, as seen in the ASO studies [13], or could be direct effects of momelotinib on these genes. In either case, this drug would be expected to have an impact on liver fibrosis and inflammation.

### 3.7. Momelotinib Reduces PNPLA3 Expression via Inhibition of the BMP Signaling Pathway

**Figure 6:**
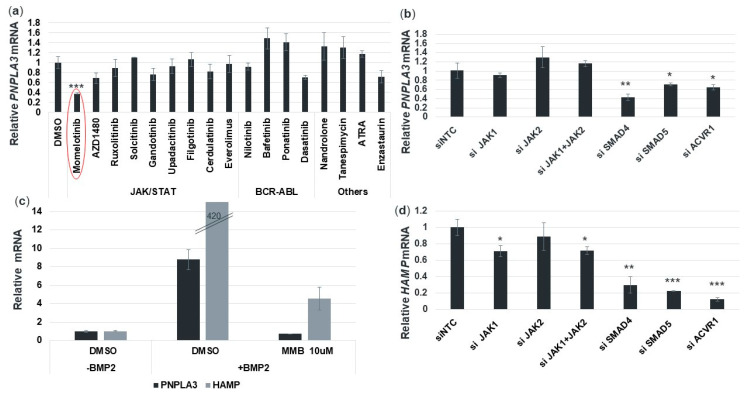
Momelotinib decreases *PNPLA3* expression primarily through SMAD pathway inhibition. (**a**) Several clinical-stage JAK inhibitors were used to treat hepatocytes for 18 h. Then qPCR was used to determine *PNPLA3* mRNA expression. The JAK inhibitor, momelotinib, was uniquely potent compared to other inhibitors in the same class. (**b**) *PNPLA3* mRNA levels after siRNA-mediated knockdown of momelotinib targets, in isolation or in combination, in human primary hepatocytes. (**c**) *PNPLA3* and hepcidin (*HAMP*) expression is strongly induced by BMP2, and momelotinib can counteract this induction. (**d**) Hepcidin (*HAMP*) mRNA levels after siRNA-mediated knockdown of momelotinib targets in human primary hepatocytes. *HAMP* expression is reduced primarily by the depletion of SMAD pathway components SMAD4, SMAD5, and ACVR1, but also is reduced by JAK silencing. For all experiments, the average of three biological replicates is shown, along with SD. One-way ANOVA was used to show significance of each siRNA treatment relative to siNon-Targeting Control (siNTC, panels **b**,**d**) or DMSO control (panel a). *, **, *** indicate *p* ≤ 0.05, *p* ≤ 0.01, and *p* ≤ 0.001, respectively.

**Figure A2:**
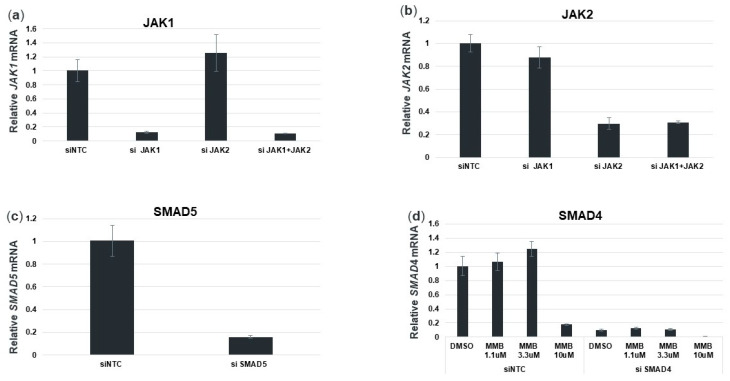
The siRNA-mediated knockdown of signaling pathway components. Pooled siRNA against JAK1 and/or JAK2 (**a**,**b**) or SMAD5 (**c**) or non-targeting control (siNTC) was used to deplete these targets in human primary hepatocytes. The qPCR analysis for the respective targets showed over 70% mRNA reduction for all. (**d**) After siRNA treatment with siNTC or siSMAD4, hepatocytes were treated with DMSO or momelotinib for 18 h prior to mRNA analysis. SMAD4 knockdown was greater than 80%. Each experiment was conducted in triplicate and average and SD are shown.

Momelotinib was developed as a selective, ATP-competitive JAK1/JAK2 inhibitor, though it also inhibits a small number of non-JAK targets [14,15]. In our initial compound screens, momelotinib was the only JAK inhibitor (out of nine) that reduced *PNPLA3* expression levels (Figure 6a). This result implied that while JAK inhibition may play a role in *PNPLA3* regulation, it is likely regulating this gene primarily via inhibition of non-JAK pathways. Recently, Asshoff et al. have demonstrated that momelotinib can also inhibit the bone morphogenic protein receptor kinase activin A receptor, type I (ACVR1) [16]. We therefore determined the dependency of *PNPLA3* on both pathways using siRNA to deplete components of JAK and ACVR1 signaling in human primary hepatocytes (Figure A2). We found that the depletion of JAK1 and JAK2, singly or in combination, had only a weak effect on *PNPLA3* mRNA levels, reducing them by no more than 10%. JAK3 is not significantly inhibited by momelotinib, nor is it expressed in primary hepatocytes. The known JAK/STAT target gene *SOCS3*, on the other hand, was reduced by 50% by JAK1 siRNA, confirming that the siRNA treatment was indeed effectively attenuating this signaling pathway (data not shown). However, knocking down components of the BMP/ACVR1/SMAD pathway produced up to 60% reductions in *PNPLA3* mRNA (Figure 6b). We conclude that this pathway plays an important role in maintaining *PNPLA3* expression. One way to test this hypothesis is by ligand stimulation of this pathway with BMP in order to activate *PNPLA3* expression. Indeed, we found that a 16 h treatment with BMP2 resulted in a 9-fold increase in *PNPLA3*. Furthermore, co-treatment with 10 μM momelotinib was able to counteract this stimulation and return *PNPLA3* to baseline levels (Figure 6c). As a control, we also measured expression of hepcidin (*HAMP*), which has been shown to be repressed by momelotinib via ACVR1 inhibition [16]. As expected, *HAMP* expression was increased dramatically by BMP2, and decreased by momelotinib treatment (Figure 6c). Like *PNPLA3*, *HAMP* was decreased by depletion of BMP/ACVR1/SMAD pathway proteins (Figure 6d). Overall, these siRNA data are consistent with the results in Figure 6a, wherein *PNPLA3* mRNA was only modestly reduced by JAK inhibitors. Momelotinib was discovered by us to be the exception, due to its ability to potently inhibit ACVR1, a key pathway controlling *PNPLA3* expression.

### 3.8. Momelotinib Reduces Chromatin Accessibility at the PNPLA3 Gene Locus

**Figure 7:**
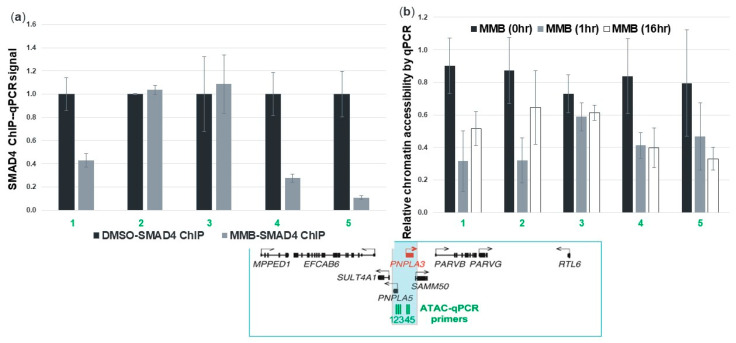
Momelotinib reduces *PNPLA3* at the level of transcription. The qPCR primers were designed to amplify peak regions of H3K27Ac and H3K4me1, two epigenetic hallmarks of active enhancer elements identified within the *PNPLA3* locus based on ENCODE datasets (shown in green). Chromatin immunoprecipitation (ChIP) for SMAD4 protein binding to predicted *PNPLA3* enhancer regions shows a dramatic loss of binding at most of these sites upon momelotinib treatment. (**a**) Assay of chromatin accessibility (ATAC) at these same enhancer regions shows reduced chromatin accessibility after 1 h and 16 h of momelotinib treatment, compared to DMSO. (**b**) Reduced accessibility at these regions is consistent with the loss of transcription factor(s) that promote *PNPLA3* transcription upon momelotinib treatment. All bars represent averages of at least three independent experiments.

The role of the SMAD family members’ involvement in transcriptional regulation has been well characterized [23]. SMAD4 acts as a convergence point for the TGFβ and BMP signaling pathways and mediates transcriptional activation within the nucleus. To determine whether momelotinib was reducing *PNPLA3* transcription via SMAD activity, we used ChIP-qPCR to examine the association of SMAD4 with chromatin before and after drug treatment. We focused specifically on a putative *PNPLA3* enhancer that we had inferred based on ENCODE ChIP-Seq data. These data provided the peak locations of H3K27 acetylation (H3K27Ac) and H3K4 monomethylation (H3K4me1), both of which are hallmarks of active enhancers. We found that SMAD4 protein could be detected at this enhancer prior to treatment, and that binding was reduced after momelotinib treatment (Figure 7a). We also used ATAC-qPCR to measure chromatin accessibility at this enhancer region using human primary hepatocytes. PCR primers were designed to amplify sequences within the enhancer that were previously observed to be accessible under normal culture conditions. We observed a reduction in accessibility after 1 h and 16 h of 10 μM momelotinib treatment, consistent with the loss of transcription factor binding in this region, supporting our observations with SMAD4 ChIP (Figure 7b). These data suggest that momelotinib exerts its effects at the level of transcription through interference of SMAD binding, though it does not formally eliminate the possibility that post-transcriptional effects also contribute.

## 4. Discussion

GWAS studies have uncovered a host of disease-associated SNPs, paving the way for the identification of novel drug targets. The Dallas Heart Study of 2008 practically revolutionized our understanding of genetic factors involved in NAFLD/NASH, pinpointing *PNPLA3* rs738409 as being strongly associated with high hepatic fat and inflammation. Several subsequent GWAS studies by other investigators confirmed and expanded on these findings across multiple ethnic backgrounds [24,25,26,27]. It is now clear that *PNPLA3* rs738409 is a gain-of-function variant, and that reducing the expression of this gene (rather than its enzymatic activity), particularly if achievable in both hepatocytes and stellate cells, is likely to be therapeutically beneficial for patients homozygous for this allele. More specifically, a 50% reduction of *PNPLA3* should be beneficial, because humans with one of two copies of *PNPLA3* 148M have an increased risk of NASH compared to non-carriers, with odds ratios of 2.35 and 5.05, respectively [6]. Further support for reducing PNPLA3 148M by at least 50% comes from a study showing that another *PNPLA3* SNP, rs2294918, reduces PNPLA3 protein levels by half, and reverses disease risk when it co-occurs with rs738409 [28].

Any NAFLD/NASH therapy aimed at reducing the *PNPLA3* 148M protein must overcome several challenges. First, the protein resides on intracellular lipid droplets, making it inaccessible to therapeutic antibodies. Second, because PNPLA3 148M protein is hypothesized to cause hepatic steatosis primarily through its toxic accumulation and sequestration of CGI-58, there are no specific enzymatic activities to be inhibited by a small molecule approach. Third, *PNPLA3* 148M expression in hepatic stellate cells is responsible for promoting fibrosis and interfering with retinol metabolism in the context of NASH and, therefore, any therapeutic agent must be able to target both hepatocytes and stellate cells. Targeting *PNPLA3* at the level of mRNA largely overcomes the first two hurdles. One strategy that has been tested in animal models is ASO-mediated knockdown of *PNPLA3* mRNA [13,22]. While this approach has indeed shown signs of efficacy, it is unclear how effectively ASOs are taken up by stellate cells. Furthermore, compared to small molecules, ASO drug development is less mature. We believe that repurposing clinical-stage drugs with well-characterized safety and pharmacological profiles represents a promising approach for treating NAFLD/NASH. Momelotinib is an excellent example, as it has the potential to overcome the aforementioned challenges for an effective *PNPLA3*-targeted NASH therapeutic, at clinically tolerable doses. As we demonstrate, it has the capability of reducing *PNPLA3* at the level of mRNA transcription and can permeate both hepatocytes and stellate cells, where it represses the activation of several profibrogenic and proinflammatory genes. The extensive body of clinical research on this particular drug positions it well for advancement into clinical trials for NASH.

The perturbation of cell signaling pathways holds great promise for treating diseases that are driven by the under- or overexpression of genes, particularly when multiple cell types must be modulated. Most genes are regulated by signaling inputs that help to fine-tune transcription levels with cell type specificity depending on environmental and developmental cues. Enhancers effectively integrate these signals by recruiting transcription factors, which are then physically transmitted to gene promoters via DNA looping [29]. The strength and frequency of these enhancer–promoter interactions influence the resultant level of transcription from a given gene. Furthermore, a single gene can be simultaneously regulated by several enhancer elements. “Decoding” the combination of signaling pathways controlling a gene is a major challenge in biology. In this study, we provide proof of principle that carefully designed signaling pathway perturbations, including both chemical and siRNA agents, can serve as tools for untangling these relationships. Furthermore, the testing of clinically validated agents alongside class members with semi-overlapping selectivity uncovers new therapeutic targets and relevant scaffolds or repurposed drug candidates. Utilizing this approach, we identified momelotinib as a potent down-regulator of *PNPLA3* transcription.

The extent to which a poly-pharmacologic drug can be used to infer gene regulation depends on the breadth of targets that are potently inhibited across a range of concentrations. Momelotinib has the benefit of inhibiting a relatively small number of kinases in the low nanomolar range [14]. However, it remained necessary to confirm these targets using a different modality. RNA interference offers the advantage of high target specificity that is lacking for most small molecules and allows for validation of pharmacologically inhibited proteins. Knocking down the mRNA encoding a target is not always equivalent to drugging the protein product of the mRNA, and the mRNA knockdown itself is generally incomplete. However, siRNAs serve as mechanistic validation, particularly when testing is concomitantly pursued against multiple targets or is combined with chemical compounds. We have used this approach to demonstrate that the BMP/ACVR1/SMAD signaling pathway is indeed responsible for maintaining *PNPLA3* expression. Overall, elucidation of these critical regulatory nodes for *PNPLA3* expression adds to our understanding of the biology of this gene and can also guide the design of new chemistries to exploit these specific mechanisms.

The strategy of leveraging signaling pathways can, in principle, be applied to any gene(s) for which there is a clear disease–driver relationship. It also offers the potential to either increase or decrease gene expression, depending on the disease hypothesis. In this study, *PNPLA3* 148M serves as an example of how reducing the expression of a disease-promoting allele by perturbing signaling pathways can have a positive phenotypic impact. Conversely, in the context of other genetically defined diseases, one might wish, for example, to upregulate the expression of a hypomorph allele to recover enzyme activity or protein function. A vast inventory of drugs has already been generated for cancer and autoimmune, inflammatory, and degenerative diseases, and many of these drugs target cell signaling kinases. At a minimum, these compounds can be used as tools to aid in the dissection of the gene regulation circuitry; at best, they may represent rapid and effective new treatment options as repurposed medicines.
